# Approved and Off-Label Uses of Obesity Medications, and Potential New Pharmacologic Treatment Options

**DOI:** 10.3390/ph3010125

**Published:** 2010-01-12

**Authors:** Maria Luisa Isidro, Fernando Cordido

**Affiliations:** Endocrine Department, Complejo Hospitalario Universitario A Coruña As Xubias 84, 15006 A Coruña, Spain; E-Mail: ma.luisa.isidro.san.juan@sergas.es (M.L.I.)

**Keywords:** obesity, drug treatment, orlistat, sibutramine, rimonabant, leptin, ghrelin

## Abstract

Available anti-obesity pharmacotherapy options remain very limited and development of more effective drugs has become a priority. The potential strategies to achieve weight loss are to reduce energy intake by stimulating anorexigenic signals or by blocking orexigenic signals, and to increase energy expenditure. This review will focus on approved obesity medications, as well as potential new pharmacologic treatment options.

## Introduction

Obesity and overweight are highly prevalent chronic conditions that are associated with premature mortality, chronic morbidity (hypertension, hyperlipidemia, type II diabetes, coronary heart disease, stroke, obstructive sleep apnea, asthma, orthopedic disorders, and certain cancers) and increased healthcare use. 

Energy balance in humans is the result of complex interactions among neuroanatomical, genetic, endocrinological, pathophysiological, nutritional, physical, psychological and social-environmental factors. Long-term maintenance of weight loss is difficult because the brain triggers compensatory physiologic adaptations that resist weight change. 

At present only two medications (orlistat and sibutramine) are approved for weight loss and weight maintenance in USA and Europe. Orlistat is a triacylglycerol lipase inhibitor that works in the intestinal lumen to reduce dietary fat absorption. Sibutramine is a serotonin-norepinephrine reuptake inhibitor that reduces appetite. Ritmonabant, a selective blocker of the cannabinoid receptor CB1 which has been shown to be involved in the central and peripheral regulation of food intake, was approved in some European countries until very recently. 

The new understanding of biology of weight regulation has provided a wide variety of potential drug targets. The potential strategies to achieve weight loss are to reduce energy intake (by stimulating anorexigenic signals or by blocking orexigenic signals) and to increase energy expenditure. It seems that the desired degree of effectiveness will more likely be achieved, with less toxicity, through the use of combinations of treatment.

## Established Therapies

The U.S. Food and Drug Administration (FDA) recommends pharmacotherapy for weight loss when lifestyle interventions (diet, exercise and behavioural therapy) have failed and the body mass index (BMI) is °30kg/m^2 ^with no concomitant obesity-related risk factors, or if the BMI is °27 kg/m^2 ^and the patient has at least one obesity-related risk factor. Adherence to lifestyle interventions (diet, exercise and behavioural therapy) should continue during pharmacological treatment. Available pharmaco-therapy options remain very limited. Once it has been established that the patient is a candidate for drug therapy, the choice of a particular drug should take into account: associated comorbidities, side effects, potential beneficial effects independent of weight loss, response, cost and the patient´s preferences. 

### Orlistat

**Figure 1:**
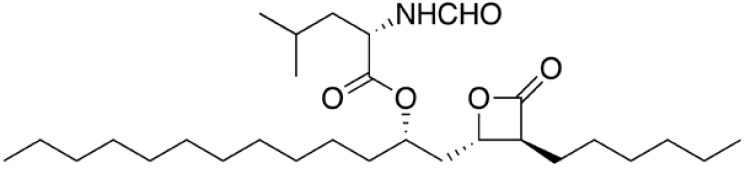
Chemical structure of orlistat.

Orlistat (Figure 1) is a gastrointestinal lipase inhibitor. It decreases fat absorption binding to pancreatic lipase, blocking hydroliyses of triglycerides into fatty acids and monoglycerides, thereby increasing faecal fat excretion by 30%. Orlistat might also modify gastric emptying and secretion of gut peptides [1], whose contribution to weight loss warrants further investigation. 

Orlistat is (*S*)-2-formylamino-4-methyl-pentanoic acid (*S*)-1-[[(2*S*,3*S*)-3-hexyl-4-oxo-2-oxetanyl] methyl]-dodecyl ester. Its empirical formula is C_29_H_53_NO_5_, and its molecular weight is 495.7. It is a single diastereomer molecule that contains four chiral centers, with a negative optical rotation in ethanol at 529 nm. 

In several randomized clinical trials (RCTs), weight loss achieved was about 3% greater for subjects taking orlistat than for those taking placebo. After one year treatment mean body weight loss was about 2.89 kg greater in the active group than in the control group [2,3,4]. A reduced incidence of type 2 diabetes was demonstrated in patients who received orlistat for four years in the Xendos study [5]. 

Due to its mechanism of action, orlistat produces significant gastrointestinal side effects (oily faecal spotting, flatus with discharge, faecal urgency, oily stools, increased defecation, faecal incontinence, abdominal pain) in 15-30% of the patients under treatment, that tend to disappear with time if the patient adheres to a low fat diet. Losses of fat-soluble vitamins have been reported with orlistat. In this context multivitamin supplementation is recommended by some authors. 

### Sibutramine 

**Figure 2:**
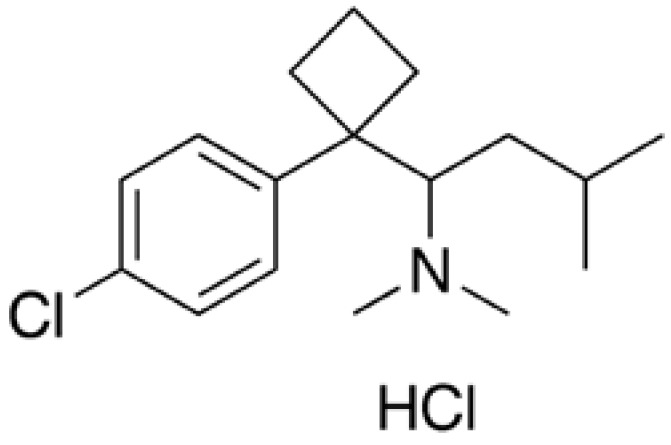
Chemical structure of sibutramine.

Sibutramine (Figure 2) is a centrally acting agent that inhibits serotonin and norepinephrine reuptake. It reduces food intake by reducing appetite. Chemically, the active ingredient is a racemic mixture of the (+) and (-) enantiomers of 1-(4-chlorophenyl)-*N,N*-dimethyl-α-(2-methylpropyl)-, cyclobutanemethanamine hydrochloride monohydrate, and has an empirical formula of C_17_H_29_Cl_2_NO Its molecular weight is 334.33. 

Long-term randomized clinical trials (RCTs) of sibutramine (10-20 mg/d) in combination with a reduced calorie diet have demonstrated modest, although significant, weight loss compared with placebo over. In several RCTs, weight loss was about 5% greater for subjects taking sibutramine than for those taking placebo [2,3,4]. 

Because of the potential to increase blood pressure and heart rate, it is recommended that these parameters are monitored in patients taking sibutramine, and its use is contraindicated in patients with uncontrolled or poorly controlled hypertension. It has been suggested that a progressive tri-therapy intervention with sibutramine-diet-exercise enhances weight loss without inducing increases in heart rate and blood pressure [6].

### Rimonabant 

**Figure 3:**
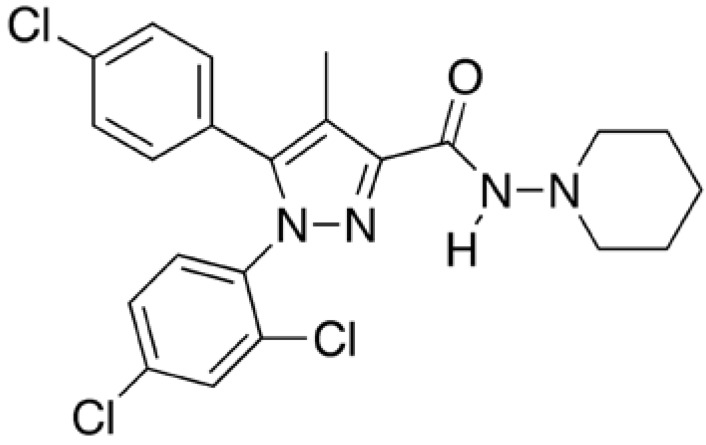
Chemical structure of rimonabant.

Cannabinoid receptors participate in the physiological modulation of many central and peripheral functions [7]. Rimonabant (Figure 3) is a selective blocker of type 1 endocannabinoid receptors that was investigated as an anti-obesity drug and for smoking cessation. It is an appetite suppressant. The FDA has not given the drug's approval because of concerns over suicide, depression and other related side effects associated with use of the drug. The European Commission approved the sale of rimonabant in the 25-member European Union, but later the drug was withdrawn from European markets because of the concerns over suicide. Chemically it is *N*-(piperidin-1-yl)-5-(4-clorophenyl)-1-(2,4-diclorophenyl)-4-methyl-1H-pyrazole-3-carboxamide. 

The degree of rimonabant intestinal absorption is unknown, but it undergoes hepatic metabolism (cytochrome 3A4 and aminohydrolase pathways) to inactive metabolites. It has biliary and faecal excretion. At present, only the results of three one-year and one two-year trials have been published. Rimonabant has shown to cause weight loss, significant improvements of cardiovascular risks factors (dyslipidemia, blood pressure and waist circumference) and reductions of HbA1c in obese type 2 diabetic patients. It is possible that rimonabant has beneficial effects on HDLc and triglyceride levels independent of weight loss, related to its direct effects on adipose tissue, liver and muscle. The most frequent side effects were nausea, dizziness, diarrhoea, insomnia and psychiatric problems (mainly depression and anxiety). Clinical trials with other CB1 receptor antagonists are ongoing [8]. Several groups are engaged in searching for novel CB1 receptor antagonists [9,10,11,12]. 

### Phentermine

**Figure 4:**
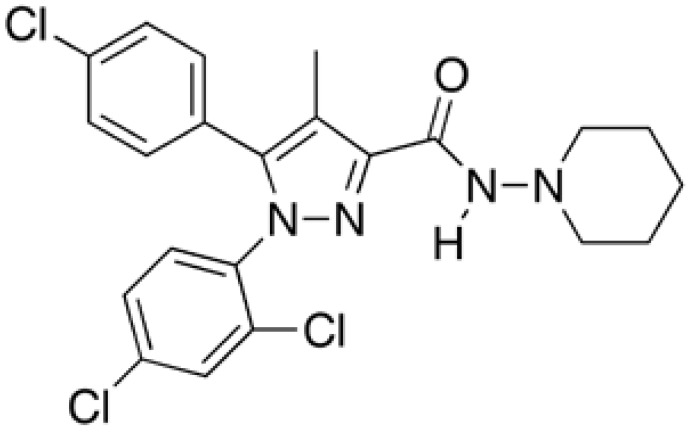
Chemical structure of phentermine.

Phentermine (Figure 4) is dimethylphenethylamine hydrochloride. It is an amphetamine-like analogue, indirectly acting sympathomimetic agents that increase norepinephrine levels in the synaptic cleft, resulting in stimulation of β_2_-adrenergic receptors and inhibition of feeding. Phentermine has also been reported to inhibit monoamine oxidase and increase the effects of serotonin, by inhibiting its pulmonary clearance. 

Phentermine is available in many countries, including the U.S. However, because it is similar to amphetamines, individuals may develop an addiction to it. Phentermine should be used short-term (usually interpreted as 'up to 12 weeks'). In the United States, it is classified as a schedule IV controlled substance under the Controlled Substances Act. Phentermine is relatively well tolerated, although it can produce side effects consistent with its catecholamine-releasing properties (tachycardia, elevated blood pressure, insomnia and restlessness). The incidence and magnitude of these appear to be less than with amphetamines. Additionally, phentermine has the potential to cause physical and psychological dependence. 

## Emerging Therapies

Because of the paucity of available anti-obesity drugs, their limited efficacy and their secondary effects, development of new and more effective drugs has become a priority. Ideally the new therapies should be more efficient, provide additional metabolic effects (beyond those attributable to weight loss), be safer, and/or have less adverse effects. 

Obesity results from a chronic energetic misbalance in which energy intake exceeds energy expenditure. Therefore, the potential targets to achieve weight loss are: (1) to reduce energy intake, by stimulating anorexigenic signals or by blocking orexigenic signals, and (2) to increase energy expenditure (Table 1). All these strategies are being investigated. 

**Table 1:** Potential antiobesity therapies.

*Drugs that stimulate anorexigenic signals:*
Leptin receptor superagonists
Peptides downstream of leptin: agonists of melanocortin receptor-4
Ciliary neurotrophic factor analogues
Agonists of 5-HT
***Drugs that inhibit orexigenic signals:***
Neuropeptide Y receptor anatagonists
Melanin-concentrating hormone-1 receptor antagonists
Somatostatin analogues
***Gastrointestinal peptides as drug targets:***
GLP-1 receptor agonists
Peptide YY 3-36 analogues
Ghrelin receptor antagonists or inverse agonists
Oxyntomodulin analogues
***Drugs that increase energy expenditure:***
Beta-adrenergic agonists
Growth-hormone receptor agonists

**Figure 5:**
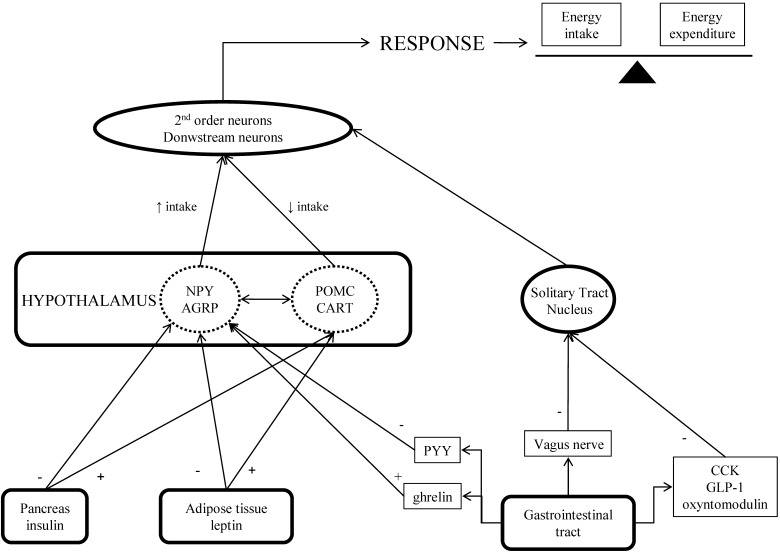
Simplified representation of control of food intake. PYY: peptide YY; CCK: cholecystokinin; NPY: neuropeptide Y; AGRP: Agouti-related protein; POMC: proopiomelanocortin; CART: cocaine and amphetamine-related transcript.

Very briefly, the hypothalamus is a primary site for the integration of several factors of central and peripheral origin for the regulation of energy homeostasis (Figure 5) [13]. The status of energy stores is conveyed to the central nervous system by adiposity-associated hormones (leptin and insulin) and possibly some gastrointestinal peptide hormones. Satiety signals from the GI tract and neuronal influences from the digestive tract, via the vagus nerve, are routed to specific nuclei of the hypothalamus and brain stem, such as the arcuate nucleus and the solitary tract nucleus, which activate the neuronal networks that control eating behavior. In the arcuate nucleus, leptin and insulin stimulate the activity of neurons that express the catabolic neuropeptide precursor proopiomelanocortin (POMC) and the anorexigenic factor cocaine and amphetamine-related transcript (CART), and inhibit neurons that produce the anabolic meditors neuropeptide Y (NPY) and the agouti-related protein (Agrp). Several of these groups of neurons are interconnected at different levels, so that activation of a group inhibits other neurons and vice-versa. Information about short-term modifications in nutrient status is conveyed to the brain through meal-related gastrointestinal hormone responses, variations in levels of nutrient content and gastric distension. This information influences the size and frequency of each individual eating episode. Except for ghrelin, that is thought to promote meal initiation, gastrointestinal signals contribute to satiation and meal termination. This feedback system, together with genetic, psychological and social-environmental factors, interacts to elicit endocrine, autonomic and behavioral answers that determine body weight. Targeting each one of these steps has pros and cons [14]. Effective treatment of obesity will probably require a combination of drugs acting at different points in this complex system. A review focused on the structural classification of the anti-obesity agents has recently been published [15]. 

## Stimulation of Anorexigenic Signals

### Leptin

Leptin is a versatile 16 kDa peptide hormone, with a tertiary structure resembling that of members of the long-chain helical cytokine family. It was originally thought to act only as a satiety factor, but there is considerable evidence for other systemic effects of leptin. At least five isoforms of leptin receptor exist, primarily because of alternate splicing. The longest form is capable of full signal transduction. The short forms may serve as leptin binding proteins and play a role in leptin transporting across the blood-brain barrier. The mechanism by which leptin modulates energy balance involves many hypothalamic neuropeptides including neuropeptide Y (NPY), the melanocortin system, melanocyte-concentrating hormone and cocaine- and amphetamine-regulated transcript [16]. 

Interest in leptin as anti-obesity drug decreased when elevated levels were noted in the majority of obese individuals [17]. Most cases of obesity are associated with leptin insensitivity or resistance, rather than leptin deficiency. Obese and non-obese subjects have similar central leptin levels, which suggest that transport to CNS, rather than intrinsic responsiveness to leptin, might be rate limiting for leptin activity in the obese state [18]. It would be necessary to develop a treatment that overcame leptin insensitivity or bypassed normal central leptin functioning, for example, by developing novel forms of leptin with stronger physiological properties [19]. The peptides downstream of leptin constitute another possible target for therapeutic interventions. Finally, another strategy would be to target genes that are involved in leptin functioning, for example, negative regulators of leptin signaling SOCS3 and PTP1B.

Literature strongly suggests that the leptin resistance is due a decreased transport of leptin across the blood-brain barrier. The main cause of this resistance appears to be an impairment in the activity of the transporter rather than just simply saturation at higher doses [20]. In fact, the transport mechanism into the brain is saturated at relatively low plasma leptin concentrations. The nose provides an effective way for delivering neuropeptides to the central nervous system, bypassing the blood-brain barrier and avoiding systemic side effects. In obesity, leptin-receptor signaling is blunted in brain areas critical to energy homeostasis, even when leptin is injected directly into the brain [21]. This problem could be addressed by creating leptin-receptor superagonists, but development of synthetic leptin-receptor agonists is in preclinical stages. 

The peptides downstream of leptin constitute another possible target for therapeutic interventions. Pro-opiomelanocortin (POMC) is the first key intermediary of leptin-receptor signaling. POMC is a complex polypeptide precursor which is cleaved into smaller biologically active peptides. Data from human genetic and murine studies show that an intact central melanocortin signaling pathway is critical for normal energy homoeostasis [22,23], but POMC-derived peptides are also involved in adrenal physiology and other functions. Cleavage of POMC produces biologically active peptides such as the melanocortins, *α*-, *β*- and *γ* -melanocyte-stimulating hormone. α-MSH activates melanocortin-3 and melanocortin-4 receptors (Mc3r, Mc4r) to exert catabolic effects. MC4R agonists specifically designed are being investigated for potential treatment of obesity. Ro-27-3225 (Bu-His-Phe-Arg-Trp-Gly-NH_2_), a nonselective human MC4R pentapeptide agonist, was reported to be able to reduce food intake and weight gain in *ob/ob* mice [24]. Using it as a template, systematic replacement of the residues was used to identify selective MC4R agonists, but the level of efficacy of these compounds was not reported. Several companies have MC4R agonists that are being investigated in the treatment of obesity, including piperazinebenzylamines, piperazinethylamines, piperazinesulfonamides and other small-molecule agonists [25]. The effects of POMC on food intake and body weight and current developments in potential therapies to manipulate this pathway have recently been reviewed [26]. 

### Ciliary Neurotrophic Factor

Ciliary neurotrophic factor (CNTF) is a 22-kDa protein that is expressed in Schwann cells in the peripheral and astrocytes in the central nervous system. The CNTF receptor complex is most closely related to the receptor complexes for interleukin-6 and leukemia inhibitory factor. Signal transduction by CNTF requires that it binds first to CNTFR alpha, permitting the recruitment of gp130 and LIFR beta, forming a tripartite receptor complex. CNTF exerts a protective effect in demyelinating disease by preventing apoptosis of oligodendrocytes. CNTF also exerts an anti-inflammatory effect in the central nervous system.

In a human study examining its usefulness for treatment of motor neuron disease, an unexpected weight loss was observed [27]. Further investigation revealed that CNTF mimics the biological actions of leptin while overcoming leptin resistance. *Axokine,* a second-generation neurotrophic factor that is related to CNTF with a 15 amino acid truncation of the C terminus and two amino acid substitutions, is three to five times more potent than CNTF in *in vitro* and *in vivo* assays, has improved stability properties and was shown to result in more weight loss than placebo [28]. Studies with Axokine were stopped due to the development of neutralizing antibodies against CNTF in a significant number of patients. This strategy as potential anti-obesity target warrants further investigation [29,30]. 

### Subtype-Selective Serotonin-Receptor Agonists 

Endogenous hypothalamic serotonin (5-HT) plays an important part in within-meal satiation and post-meal satiety processes, apart from in several sensory, motor and behavioral processes. Numerous serotonin receptor subtypes have been identified; of these, serotonin 5-HT2C and 5-HT1B receptors have been specifically recognized as mediators of serotonin-induced satiety [31,32,33]. Activation of 5-HT2C receptors on arcuate POMC neurons engages the same melanocortin pathway that is critical to leptin-mediated anorexia. 5-HT1B activation on arcuate NPY/Agrp cells inhibits neuronal activity, resulting in indirect stimulation of POMC cells, complementing the direct activation of the same neurons by the 5-HT2C receptor. This effect lies downstream of some of the levels at which leptin resistance occurs in obesity. Thus, the serotonin system has provided a viable target for weight control [34].

A small number of short-term studies using isoform-selective 5-HT agonists confirm that stimulation of 5-HT2C receptor, and possibly 5-HT1B receptor, reduces food intake and weight in humans. A combined 5-HT2C/1B agonist (*m*-chlorophenylpiperazine) and the selective 5-HT2C agonist lorcaserin [(1*R*)-8-chloro-2,3,4,5-tetrahydro-1-methyl-1*H*-3-benzazepine, APD356] have been tested in obese individuals, with modest but significant results. Several 5-HT2C selective agonists are under development [35]. In addition, 5-HT6 receptor antagonists such as PRX-07034 and BVT74316 have been shown to reduce food intake and bodyweight gain in rodent models and have recently entered clinical trials [36,37,38]. 

## Inhibition of Orexigenic Signals 

### Neuropeptide Y Receptor Antagonists

Neuropeptide Y (NPY) is a widely distributed peptide in the central nervous system of both rodents and humans [39]. It has been implicated in a variety of physiological actions, including control of body weight. In mammals, the signaling is mediated via at least five different cell surface receptors, denoted as Y(1), Y(2), Y(4), Y(5) and Y(6). There is no consensus regarding which subtype is the most important for NPY-induced feeding and attempts to demonstrate an important role for NPY in the control of food intake has produced equivocal results. Antagonists of the NPY Y(1) and NPY Y(5) receptor subtype initially looked promising. However, attempts to inhibit the signaling of NPY through the NPY Y(1) and NPY Y(5) receptors has produced equivocal effects on food intake, and clinical studies of Y-receptor antagonists are almost nonexistent at present. 

### Melanin-Concentrating Hormone Antagonists

Melanin-concentrating hormone (MCH) is a cyclic nonadecapeptide, that is abundantly present in mammalian neurons. MCH binds to and activates two G protein-coupled receptors, MCH1R and MCH2R. The MCH-1 receptor (MCH-R1) has been identified as a key target in MCH regulation. In addition to the crucial roles of MCH in feeding behaviour, anatomical and neurochemical studies suggest that the MCH/MCH(1) system is involved in the regulation of emotion and stress responses. Therefore, it is important to develop anti-MCH agents that selectively modulate energy homeostasis without exerting other side effects. 

Multiple chemotypes of small molecule MCHr1 antagonists have been identified and shown to induce weight loss in animal models [40-42], but many of these lead compounds have been found to cross-react with the hERG potassium channels (channels encoded by the human ether-a-go-go-related gene), which are involved in cardiac action potential repolarization, and/or demonstrate deleterious effects on cardiovascular hemodynamic parameters. 

### Somatostatin Analogues

Somatostatin and its analogues (octreotide and lanreotide) bind to somatostatin subtype 5 receptors on the beta-cell membrane, which limits insulin release and, consequently, may decrease adipogenesis. Long-acting release octreotide was used in hyperinsulinaemic obese adults and resulted in statistically significant weight loss [43,44]. The patients with the greater degree of insulin hypersecretion appeared to derive the most benefit from treatment. 

## Gastrointestinal Peptides That Regulate Food Intake, As Drug Targets

Information about short-term changes in plasma levels of certain nutrients are communicated to the brain through gastrointestinal peptides, acting in conjunction with information about gastric distension, via the vagal and spinal nerves [45-47]. Except for ghrelin, gastrointestinal signals contribute to satiation. Individual peptides are not secreted in isolation in response to nutrient ingestion. Rather, there is a coordinated release of several hormones that act in coordination with CNS reward pathways, input from higher centers and social and environmental influences. This short-acting GI signals are processed in the central nervous system, along with information about the status of the body energy stores, to elicit corresponding alterations in catabolic and anabolic neuropeptides and neurotransmitters to control energy homeostasis. To increase the efficacy of anti-obesity drugs, it will probably be necessary to develop combination agents that target multiple signals in the energy homeostasis system. 

The theoretical advantage of reproducing the body´s own satiety signals would be that ubiquitous neurotransmitter systems would be minimally disturbed and undesirable side effects would be expected to be reduced. One issue that limits the use of native peptides is their short half-life, which conditions inconvenient administration regimes. The development of stable analogues and novel methods of drug delivery are crucial parts of drug development. 

Gastrointestinal peptides that regulate food intake include glucagon-like peptide-1, peptide YY3-36, oxyntomodulin (OXM) and ghrelin, among others. Gut hormones as potential new targets for appetite regulation and treatment of obesity have recently been reviewed [48]. 

### Glucagon Like Peptide (GLP-1) Receptor Agonists

Pre-proglucagon derived peptides Glucagon-Like Peptide-1 (GLP-1), Glucagon-Like Peptide-2 (GLP-2) and oxyntomodulin (OXM) are involved in a wide variety of physiological functions. The major physiological role of GLP-1 in mammals is to connect the consumption of nutrients with glucose metabolism [49]. To date, clinical development has focused on its incretin effect (intestinal enhancer of insulin secretion) and its use as antidiabetic agents. Peripheral administration of GLP-1 derivatives and analogues to both rodents and man has shown to have effects on food intake and body weight, by inducing satiety and decreasing food intake. In young healthy subjects GLP-1 infusion decreases spontaneous energy intake and *ad libitum* hunger, suggesting that GLP-1 may play a physiological regulatory role in controlling appetite and energy intake in humans [50,51,52]. The therapeutic utility of the native GLP-1 molecule is limited by its rapid enzymatic degradation by a serine protease termed dipeptidyl peptidase-IV (DPP-IV). A number of DPP-IV-resistant GLP-1 agonists, including exenatide and liraglutide, have been developed. Exenatide, or exendin-4, (C_184_H_282_N_50_O_60_S^.^C_2_H_4_O_2_), was extracted from the venom of the gila monster; it is supplied for subcutaneous (SC) injection and marketed to treat diabetes, and causes a modest but progressive weight lost. Liraglutide Arg(34)Lys(26)-(*N*-ε-(γ-Glu(*N*-α-hexadecanoyl))-GLP-1(7-37) was synthesized using the GLP-1 sequence with the addition of an acyl side chain that allows for noncovalent binding to albumin, which prolongs its half-life in the circulation. In trials evaluating efficacy of incretin therapy in type 2 diabetes that reported data on changes in weight, there was a statistically significant weight loss observed with GLP-1 analogues versus comparator groups. Although GLP-1 receptor agonists are not currently approved for obesity treatment, it is possible that they have a role as an anti-obesity treatment [53]. 

### Peptide YY Analogues

PYY3-36 is the major form of circulating PYY and binds to the hypothalamic Y2 receptor. PYY is hypothesised to inhibit food intake via activation of the auto-inhibitory presynaptic NPY Y2-R present on the NPY neurons located in the arcuate nucleus, and activating adjacent anorexigenic POMC neurons [54,55]. The role of oxyntomodulin and peptide tyrosine-tyrosine (PYY) in appetite control has recently been review [56,57]. It has been suggested that disregulation in the secretion of this anorexigenic peptide may contribute to the complex pathogenesis of anorexia of some diseases, such as chronic renal failure [58]. Effects on other processes affecting energy balance (energy expenditure, fuel partitioning, gut nutrient uptake) remain poorly understood. Besides energy balance, PYY has been shown to coordinate gastrointestinal functions and has some role in other systemic functions such as control of blood pressure, heart rate and sleep. 

PYY3-36 has a functional half-life of approximately 3 h. Attachment of poly(ethylene glycol) (PEG) and coupling it to a 40 kDa PEG through a spontaneously cleavable linker develops a reversible PEGylated PYY3-36 derivative, and results in an eightfold increase in its functional half-life, to approximately 24 h. Variability of its effect across different experimental conditions in animal models led to confusion on its potential as an anti-obesity treatment. Some studies suggest that PYY has, if at all, only a minor role in food intake in rats, but a number of groups has demonstrated that peripheral PYY3-36 inhibits food intake and reduces body weight gain in other species. Pattern of administration is critical for producing a sustained effect of PYY3-36 on food intake and adiposity in rodents. The pharmacological value of PYY is controversial. Further studies are indicated to determine the potential role in energy balance regulation, and the optimal delivery and dosing. 

### Ghrelin Receptor Antagonists and Inverse Agonists

Ghrelin is the only known gut orexigenic hormone. It is an endogenous ligand for GH secretagogue 1A recertor (GHS_1A_-R). Apart from other actions, it seems to have a role in meal initiation and long-term control of body weight [13]. We have studied the potential relationships between ghrelin and malnutrition in some chronic diseases [59,60]. Ghrelin levels are low in obese individuals and rise in response to weight loss, as a compensatory response to promote weight regain. This could suggest that disruption of ghrelin signaling would not be useful in treating obesity. Several factors indicate that this idea might be incorrect: It is possible that obese individuals are more sensitive to the orexigenic effects of ghrelin [61] and obesity is associated with an attenuation of the post-prandial ghrelin fall [62]. It is also possible that ghrelin blockage would be more useful in preventing weight regain, after weight loss is achieved by other means. 

Highly potent GHS_1A_-R antagonists have been identified [63,64,65,66]. In rat models, some but not all GHS_1A_-R antagonists decreased food intake and body weight when administered centrally or intraperitoneal [67,68]. The GHS_1A_-R has constitutive activity [67] and, therefore, inverse GHS_1A_-R agonists [69] may prove to be more effective in inducing weight loss than GHS_1A_-R antagonists. Other more innovating approaches to decrease ghrelin activity have also been investigated as potential treatments [70,71,72,73].

### Oxyntomodulin Analogues

Oxyntomodulin is a 37-amino-acid peptide that contains the 29-amino-acid structure of glucagon, followed by an octapeptide *C*-terminal extension [74]. It has been suggested that oxyntomodulin exerts its anorectic effect by signaling through the GLP-1 receptor. The administration of oxyntomodulin, when given intraperitoneally or into the cerebral ventricles, has been observed to reduce short-term food intake in rodents [75,76]. Oxyntomodulin-treated animals lose more weight than control animals that consume the same amount of calories, which suggests that oxyntomodulin increases energy expenditure, possibly via an effect on the thyroid axis. Oxyntomodulin has been found to reduce energy intake in normal-weight volunteers when administered intravenously or subcutaneously before a single study meal [77,78,79]. This weight-loss effect in humans could be caused by an increase in energy expenditure, in addition to a decrease in energy intake, as previously suggested by rodent data. 

## Drugs That Increase Energy Expenditure 

There are many potential targets to stimulate energy expenditure or alter substrate utilization [80]. Increased energy expenditure involves either increased ATP utilization or oxidation of reduced coenzymes by enzymes or pathways that are not coupled to ATP. Thermogenesis may be initiated centrally or may be a direct peripheral action. Peripherally acting thermogenic drugs seem less likely to have side effects than centrally acting drugs and may have additional metabolic benefits.

### β3-Adrenergic Agonists

Treatment of obese animals with *β*3-adrenergic agonists increase lipid mobilization, induce mitochondrial uncoupling protein-1 and, finally, reduce body fat content. All weight loss is lipid, and lean may actually increase. The main sites of action *β*3-adrenergic agonists are white and brown adipose tissue, and muscle. *β*3-Adrenoceptor agonists fall into two main chemical classes: arylethanolamines and aryloxypropanolamines. It is difficult to identify *β*3-adrenoceptor agonist drugs because of differences in pharmacology between the rodent and human *β*3*-*adrenoceptors [81,82]. Moreover, near absolute selectivity is needed to avoid *β*(1/2)-adrenoceptor-mediated side effects and selective agonists tend to have poor oral bioavailability. Several phase II trials with this type of drugs were discontinued because of poor drug efficacy [83,84] and safety profiles. 

### Growth Hormone

Human growth hormone (GH) has profound lipolytic/antilipogenic actions in vivo and its secretion is decreased in obesity [85,86]. A recent meta-analysis of human studies examining the efficacy and safety of recombinant GH as therapy for obesity [87] concluded that rhGH therapy leads to decrease in visceral adiposity and increase in lean body mass without inducing weight loss. The rhGH doses used in many studies were supraphysiologic, and the authors suggested that future studies of longer duration, using carefully titrated rhGH protocols, will be needed in order to fully establish the effects of rhGH therapy in obesity. 

Growth hormone receptor agonists** could be another potential target for obesity treatment. A small region of the growth hormone molecule, denoted hGH 177-191, appears to retain some of the actions of growth hormone, but with no effect on growth or on insulin resistance. An orally active peptide variant of hGH 177-191, called AOD9604, was shown to stimulate metabolism of fat in animal trials [88,89] but, after a phase IIB clinical trial, the results did not support the commercial viability of the drug as a treatment for obesity. 

### Other Thermogenic And Metabolic Drugs

There are many potential ways to stimulate energy expenditure or alter substrate utilization, but it is somewhat premature to discuss some of them, such as mitochondrial uncoupling [90], as targets in the treatment of obesity. 

## Anti-Obesity Drugs And Safety Concerns

Obesity and overweight are highly prevalent chronic conditions that are associated with premature mortality, chronic morbidity and increased healthcare use. At the same time, there is a real potential for widespread misuse of this kind of drugs. It is essential to develop effective but, at the same time, safe and well tolerated new agents. Previous medicines, particularly centrally acting agents, have poor safety records and safety concerns have led to the withdrawal of some of them [91,92]. Preliminary data have very recently aroused safety concerns with sibutramine [93]. Although the official results are not yet available, this may have implications for the future approval of drugs that act through the same mechanism of action, like tesofensine [94], and even the strictness of the approval criteria of any new agent. 

The identification of a potential target is a long way from the synthesis of a compound that might become a drug. Issues of bioavailablity, metabolism, clearance, interactions and toxicity have to be addressed. Translatability of the pharmacological effects from animals to humans is challenging with anti-obesity drugs. There is no good way to predict rare or apparently human-specific side effects, apart from conducting large clinical trials and extensive monitoring efforts after market release. In 1996, the FDA established draft guidelines with recommendations for the design and conduct of clinical studies evaluating weight control drugs. 

## Conclusions 

Studies evaluating the long-term efficacy of anti-obesity agents are limited to orlistat and sibutramine. Both drugs appear modestly effective in promoting weight loss; however, interpretation is limited by high attrition rates. Longer and more methodologically rigorous studies of anti-obesity drugs that are powered to examine endpoints such as mortality and cardiovascular morbidity are required to fully evaluate any potential benefit of such agents. Orlistat is potentially preferred in the presence of diabetes, dislipidemia, hypertension or cardiovascular disease, in the absence of gastrointestinal disease; sibutramine is the drug of choice when lack of satiety is a major barrier to weight reduction, in the absence of cardiovascular disease.

Development of new and more effective drugs has become a research priority. The potential strategies to achieve weight loss are: (1) to reduce energy intake, by stimulating anorexigenic signals or by blocking orexigenic signals, (2) to increase energy expenditure. All these strategies are being actively investigated, although it is not probable that a solution will be available in the near future. The new drugs should take into account these pathophysiological pathways. We believe that a drug that acts through the gastrointestinal peptides that regulate food intake, like a ghrelin antagonist or a GLP-1 agonist, could be good options. The desired degree of effectiveness will more likely be achieved through the use of combinations of treatments.
